# Microblog-HAN: A micro-blog rumor detection model based on heterogeneous graph attention network

**DOI:** 10.1371/journal.pone.0266598

**Published:** 2022-04-12

**Authors:** Bei Bi, Yaojun Wang, Haicang Zhang, Yang Gao

**Affiliations:** 1 College of Information and Electrical Engineering, China Agricultural University, Beijing, China; 2 School of Computer Science and Technology, Beijing Institute of Technology, Beijing, China; 3 Key Lab of Intelligent Information Processing, Institute of Computing Technology, Chinese Academy of Sciences, Beijing, China; 4 Zhongke Big Data Academy, Zhengzhou, Henan, China; 5 School of Economics and Management, Beijing University of Technology, Beijing, China; Hanyang University, REPUBLIC OF KOREA

## Abstract

Although social media has highly facilitated people’s daily communication and dissemination of information, it has unfortunately been an ideal hotbed for the breeding and dissemination of Internet rumors. Therefore, automatically monitoring rumor dissemination in the early stage is of great practical significance. However, the existing detection methods fail to take full advantage of the semantics of the microblog information propagation graph. To address this shortcoming, this study models the information transmission network of a microblog as a heterogeneous graph with a variety of semantic information and then constructs a Microblog-HAN, which is a graph-based rumor detection model, to capture and aggregate the semantic information using attention layers. Specifically, after the initial textual and visual features of posts are extracted, the node-level attention mechanism combines neighbors of the microblog nodes to generate three groups of node embeddings with specific semantics. Moreover, semantic-level attention fuses different semantics to obtain the final node embedding of the microblog, which is then used as a classifier’s input. Finally, the classification results of whether the microblog is a rumor or not are obtained. The experimental results on two real-world microblog rumor datasets, Weibo2016 and Weibo2021, demonstrate that the proposed Microblog-HAN can detect microblog rumors with an accuracy of over 92%, demonstrating its superiority over the most existing methods in identifying rumors from the view of the whole information transmission graph.

## 1. Introduction

In recent years, a microblog has become a widely used social media platform based on a user’s desire to share, spread, and acquire short real-time information. Microblog rumors represent posts that spread through a microblog and are confirmed as false information in the process of transmission. In general, they are characterized by rapid diffusion, broad reach, and great harm. When hot social issues are involved, rumors can easily incite negative emotions of net citizens, disrupt social harmony, and even destructively weaken the authority of public departments. In addition, companies drawn into rumors face a disturbance in the stock price and regular operation, while public figures who have become the protagonists of rumors suffer from excessive attention on personal and family affairs. Therefore, early identification and clarification of microblog rumors are significant in monitoring online public opinion and maintaining social stability.

At present, the microblog service providers recognize rumors by handling tip-offs, which has a high detection cost and time lag. However, if a model could be designed to detect rumors automatically and give a timely warning to credible departments to undertake a further investigation, the workload and time-consumption of rumor detection would be significantly reduced, and the negative effect of rumors would be controlled. In the early years, researchers manually extracted microblog features from the microblog content and user profiles and fed them to the input of traditional machine learning classifiers, such as random forest [1] and support vector machine (SVM) [2]. With the development of deep learning, a number of detection methods based on the recurrent neural network (RNN) [3] and convolutional neural network (CNN) [4] have emerged. Still, these methods seldomly consider the multimedia form of a microblog and fail to explore the semantic information of microblog information’s spreading path fully.

Recently, modeling the information network as a heterogeneous graph has been a novel way to make the most of structure information. The heterogeneous graph can be defined as a graph model consisting of more than one type of node and edge. Different types of nodes are allowed to present different dimensional feature vectors. Specifically, a heterogeneous graph neural network has been constructed to process heterogeneous graph data. This network has been a popular algorithm currently and has been successfully applied to the fields of biomedicine [5], human-computer interaction [6], and network security [7]. When combined with an attention mechanism, a heterogeneous graph attention network encourages nodes to focus on important neighbors and can achieve superior performance on multiple public datasets from different domains [8]. Inspired by this work, this study proposes a rumor detection model for Sina Weibo, which is based on the analysis of post content and communication network.

The contributions of this study can be summarized as follows. First, the information transmission network of a microblog is modeled as a weighted heterogeneous graph, and three meta-paths whose semantic information contributes to rumor detection are introduced. Second, the multimedia information form of a microblog is processed, and the textual and visual features are extracted and grouped for later classification. Third, a rumor detection model based on a heterogeneous graph attention network with two attention layers is proposed to learn representations of posts using structural and semantic information. The node-level attention layer fuses the information of important neighbors, while the subsequent semantic layer aggregates different semantic data to form a final node embedding. Finally, a series of experiments are conducted on two real-word Sina Weibo rumor datasets, of which we crawled the second most recent experimental dataset. The Sina Weibo is used to verify the proposed model because it has been the leading microblog service provider with the most significant active users in China. The experimental results verify the superiority of the proposed Microblog-HAN model over the most existing rumor detection models, as well as the contribution of three meta-paths.

The rest of the article is organized as follows. Section 2 presents the literature review of rumor detection models and graph neural networks. Section 3 describes the modeling process of the heterogeneous graph of microblog information diffusion and the construction process of the rumor detection model using convolution layers and a heterogeneous graph attention network. Section 4 presents experimental results on two real annotated microblog rumor datasets. Finally, Section 5 draws the conclusions.

## 2. Related work

### 2.1 Rumor detection methods

Rumor detection is an information authenticity classification task to determine whether a given message on social media is true or fake [9]. The detection models used in the early studies denoted traditional machine learning-based classifiers. For instance, the decision tree and random forest methods have been used to detect the authenticity of posts on Twitter [1, 10, 11]. These methods typically first extract features from the posts, users, and communication network and then use the extracted features as input of the tree classifier to realize rumor detection. Ma et al. [12] built a propagation tree based on the propagation structure of Twitter posts and revealed differences between the propagation trees of rumor and non-rumor data. The propagation tree kernel method was used to classify the propagation tree and determine whether a Twitter post had been a rumor or not. In addition, Ma et al. [13] considered the time characteristics of rumors, employed the time series modeling approach to integrate various rumor information, and used an SVM classifier as a rumor detection model. Further, Yang et al. [2] extracted 19 types of features from the Sina Weibo dataset, which were then used as the SVM input; these features originated from microblog content, comments, and user avatar types. Some of the extracted features have been specific to Weibo, such as the geographic location of events and the client program used to publish the microblog. The experimental results show that specific features impose a different degree of influence on the prediction effect of a microblog and Twitter.

Moreover, with the development of deep learning, Ma et al. [3] first used a neural network model to detect rumors. They applied an RNN model to learn the context information of online rumors and revealed that the RNN outperformed other methods based on handcrafted features on Twitter and Weibo datasets. Also, Ma et al. [14] investigated the non-sequential propagation structure following the content of tweets, established propagation tree nodes according to the commenters’ orientation, and proposed two recursive neural network models based on the bottom-up and top-down tree-structured neural networks. Guo et al. [15] integrated the attention mechanism with an RNN and assigned various attention weights to different text features, thus achieving better classification performance. Jin et al. [16] also leveraged the attention mechanism based on an RNN, which merges image features into the combined features of text and social context extracted from posts. This model can learn the multi-modal features of rumors.

A CNN has also been a widely used deep learning-based method in the field of rumor detection. Yu et al. [4] first applied a CNN to conduct the rumor detection and achieved good results. The convolutional layer was used to learn the critical features of the input microblog text and comments sequences, and then the higher-level interaction between the features was obtained. Liu et al. [17] combined the RNN and CNN to construct a classifier that can process time-series information, capturing the global and local variations of users’ characteristics along the transmission path. Considering a significant emotional tendency of rumors, Lv et al. [18] constructed a rumor detection model based on the CNN-LSTM combination, treating a comment sentiment as an important feature for rumor detection. A generative adversarial network could solve some of the complex problems, which could improve classifier robustness. Ma et al. [19] adopted an adversarial learning method, where the generator learns the realistic microblog and produces more misleading training materials by distorting non-rumors and whitewashing rumors, thus forcing the discriminator to learn more robust rumor representations.

The research on automatic microblog rumor detection has still been in its infancy. Traditional machine learning-based methods have common defects of high time consumption and laborious manual feature extraction. Moreover, the extracted features often lack a high-level representation of microblogs, which results in the loss of potential information and inaccurate detection of rumors. In contrast, deep learning-based models can avoid tedious feature engineering and automatically learn the advanced features of rumors, further improving the accuracy of rumor detection. However, the existing methods generally fail to address the global relationship of information transmission graph or tree, thus unsuccessfully making full use of the spread information of a microblog. Moreover, little consideration has been given to the multi-modal contents other than text, such as pictures, audio, and videos.

### 2.2 Graph neural network

A graph neural network (GNN) is a neural network model specially designed for graph data, which can handle classification and regression tasks at the node, edge, and graph levels. Sperduti [20] first applied an RNN to a directed cyclic graph to analyze the complex graph. Gori et al. [21] proposed a GNN model that can directly process graph data. The feature of each graph node was determined by the node itself and its neighboring nodes. Based on this principle, the node representation could be learned iteratively. This model performs well for both undirected and directed graphs. Inspired by CNNs, researchers extended convolution operations to the graph data and constructed a graph convolution network (GCN). Bruna et al. [22] defined the graph convolution operation based on spectral graph theory. However, spectral convolution operations required processing the whole graph simultaneously during the calculation, so the time complexity was too high. To address this limitation, Mikael et al. [23] developed a spatial-based GCN, which can significantly reduce the amount of computation by performing the convolution operation directly on the graph structure.

A graph attention network (GAT) is another type of the GNN. In the sequence-based tasks, the attention mechanism can learn the importance of each part of a sequence. The basic idea of GAT is to compute the hidden node representations by attending over the neighbors. The first GAT was designed by Velikovi et al. [24], which assigns different attention coefficients to adjacent nodes and fuse them without costly matrix operations. Zhang et al. [25] further elaborated the model, adopting a more stable multi-attentional mechanism when updating the hidden features of nodes, and a convolutional subnetwork was developed to control the importance of each attention head. Nevertheless, this model is suitable only for isomorphic graphs containing only one node and edge. However, in practice, a far more common data structure is a heterogeneous graph with multiple nodes and edges. Through exploring how to apply attention mechanisms to heterogeneous graphs, Wang et al. [8] developed a heterogeneous graph attention neural network (HAN). Aiming at the shortcomings that the GCN and GAT cannot take advantage of edges’ characteristics, Gong et al. [26] proposed a new framework that can integrate the existing graph neural networks and enable edge features to participate in training.

## 3. Method

By denoting posts, users, comments, and reposts as nodes and actions of posting and responding as edges, this study designs a heterogeneous graph of a microblog, which represents an input of a Microblog-HAN. The proposed model includes two main parts. The first part extracts the initial textual and visual features of posts, and the second part represents a modified HAN [8] with a hierarchical attention mechanism, including the node-level and semantic-level attention mechanisms and performing the node classification task.

### 3.1 Microblog information propagation graph

Microblog has been a widely used social media platform whose information transmission network can be modeled as a heterogeneous graph [27]. The heterogeneous graph of a microblog contains at least two types of nodes, namely, a post and a user who posted the post; repost and comment actions can be viewed as nodes in the heterogeneous graph if there are some responses.

In the heterogeneous graph of a microblog, nodes are connected via three types of edges: User-Post, User-Comment/Repost, and Post-Comment/Repost. Generally speaking, a post posted by a user reflects the user’s inner will, so the weights of the User-Post and User-Comment/Repost edges can be set to one. The weight of a Post-Repost/Comment edge is defined as a support degree of the repost or comment to the post. Except for those reposts or comments whose content is "Repost" or "Forward quickly", the stance of responses can be inferred by sentiment analysis. The position of a commenter has often been embodied by whether the emotional polarity of the comment is similar to that of the post. Thus, the support of a comment or repost to the corresponding post can be quantified by the difference in the emotional score of the text between them. Assume the emotional score of a post *s* is denoted by *g*_*s*_, and the counterpart of comment *c* is denoted by *g*_*c*_; then, the support of the comment *c* to the post *s* can be defined as

$$f_{sg}=1‐|g_{s}‐g_{c}|.$$ (1)

**Fig 1:**
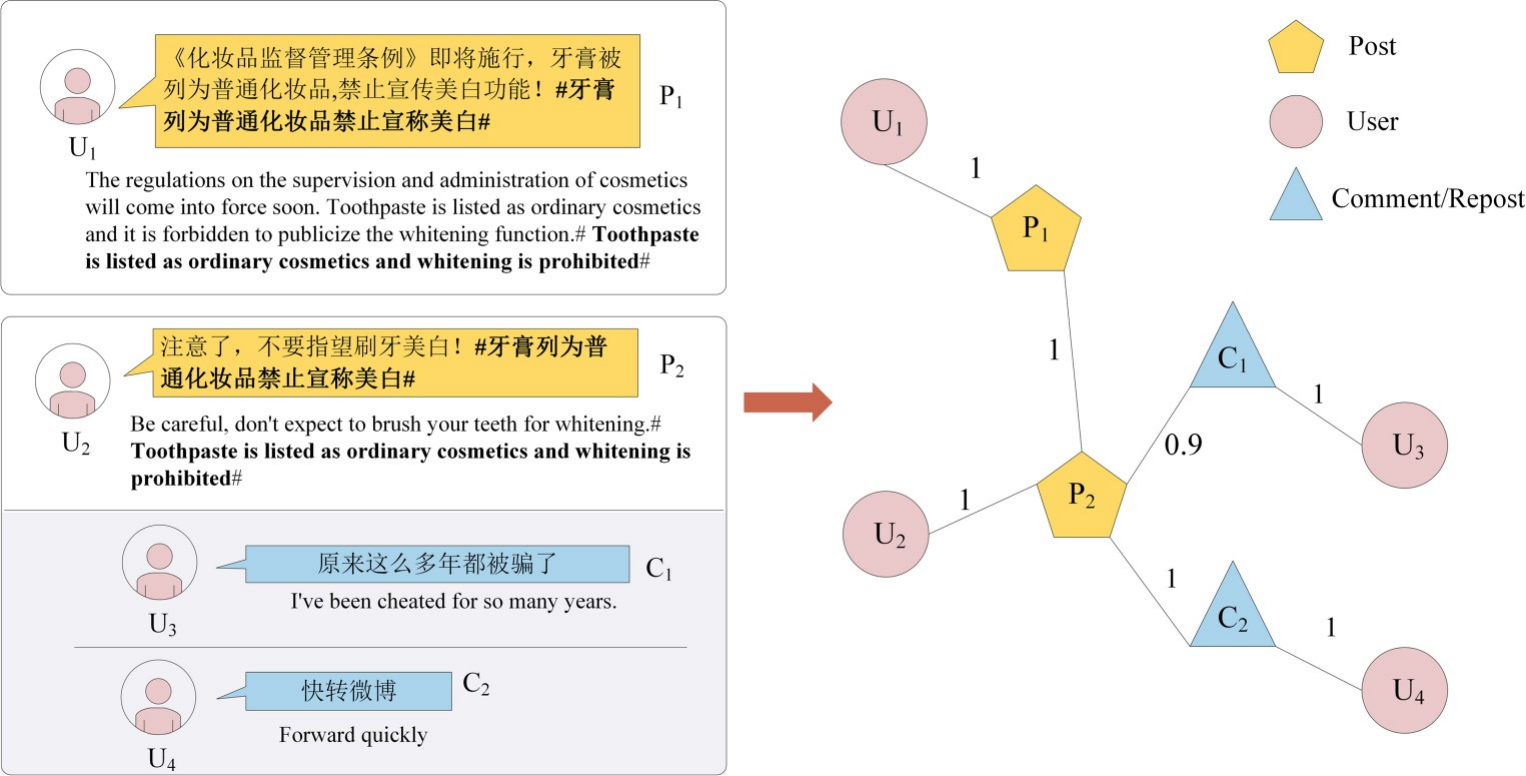
The heterogeneous graph of a microblog.

If two posts contain the same topic, the content is framed by two ’#’s, an edge is established between these two nodes, and the weight of the edge is set to one. An illustration of the microblog modeling process is shown in Fig 1. Two content-related posts, one with no comments and the other one with two followers, are constructed into a heterogeneous graph.

### 3.2 Preliminary analysis

#### 3.2.1. Meta-path description

A heterogeneous graph is composed of multiple meta-paths [28]. A meta-path Φ can be defined as $A_{1}\overset{R_{1}}{\to }A_{2}\overset{R_{2}}{\to }\ldots \overset{R_{l}}{\to }A_{l+1}$, which can be simplified as *A*_*1*_*A*_*2*_…*A*_*l+1*_, and it describes a compound relationship *R = R*_*1*_*°R*_*2*_*°…°R*_*l*_ from node *A*_*1*_ to node *A*_*l+1*_, where ∘ indicates the composition operator.

In the heterogeneous graph of a microblog, post nodes can be linked via multiple meta-paths, and different meta-paths can contain different semantic information. In this study, three types of meta-paths are considered: PP (Post→Post), which indicates that two posts share the same topic, and PUP (Post→User→Post) and PCUCP (Post→Comment/Repost→User→Comment/Repost→Post), which indicate that two posts are published and responded to by the same user, respectively.

#### 3.2.2. Meta-path-based neighbors

Given a meta-path Φ, the meta-path-based neighbors of node *i* can be defined as a set of nodes connected to the node *i* via the meta-path Φ [8], which is denoted by *N*_*i*_^*Φ*^. In particular, the neighbors of a node include itself. For instance, consider a heterogeneous microblog graph and assume that a user *U*_*j*_ publishes a microblog *P*_*i*_; then, the neighbors of the microblog (i.e., node) *P*_*i*_ based on the meta path PUP constitute a node-set of all posts published by the user *U*_*j*_, including the microblog *P*_*i*_ itself.

#### 3.2.3. Meta-path-based weight

Given a symmetric meta-path Φ that connects more than two nodes, a compound relationship of Φ can be expressed as *R = R*_*1*_*°R*_*1*_ or *R = R*_*1*_*°…°R*_*n*_*°R*_*n*_*°…°R*_*1*_ (*n* > 1); *r*_*k*_ is the edge feature of *R*_*k*_. Assume nodes *i* and *j* are linked via the meta-path Φ; then, the meta-path-based weight between the node *i* and node *j* is defined by $s_{ij}^{\Phi }=1-|\sum_{k=1}^{n}r_{k}-\sum_{l=n+1}^{2n}r_{l}|$. In other words, the relevance of node *i* and node *j* is quantified by the difference in the total weights between two halves of the meta-path. When multiple paths between node *i* and node *j* meet the form of meta-path *Φ*, the meta-path-based weight of each path is averaged to yield $s_{ij}^{\Phi }$, that is, $s_{ij}^{\Phi }=1-Avg(\left|\sum_{k=1}^{n}r_{k}-\sum_{l=n+1}^{2n}r_{l}\right|)$ Assume a microblog, where post *P*_*i*_ and post *P*_*j*_ are commented by users *U*_1_ and *U*_2_; then, the weight based on the meta-path PCUCP of post *P*_*i*_ and post *P*_*j*_ can be calculated by $s_{ij}^{\Phi }=1‐(\left|f_{i1}‐f_{j1}|+|f_{i2}‐f_{j2}\right|)/2$, where $\left|f_{i1}-f_{j1}\right|$ and $\left|f_{i2}-f_{j2}\right|$ represent the disagreement values of users *U*_1_ and *U*_2_ on posts *P*_*i*_ and *P*_*j*_, respectively. The larger the value of *s*_*ij*_^Φ^ is, the closer users’ attitudes towards *P*_*i*_ and *P*_*j*_ are.

### 3.3 Proposed model

**Fig 2:**
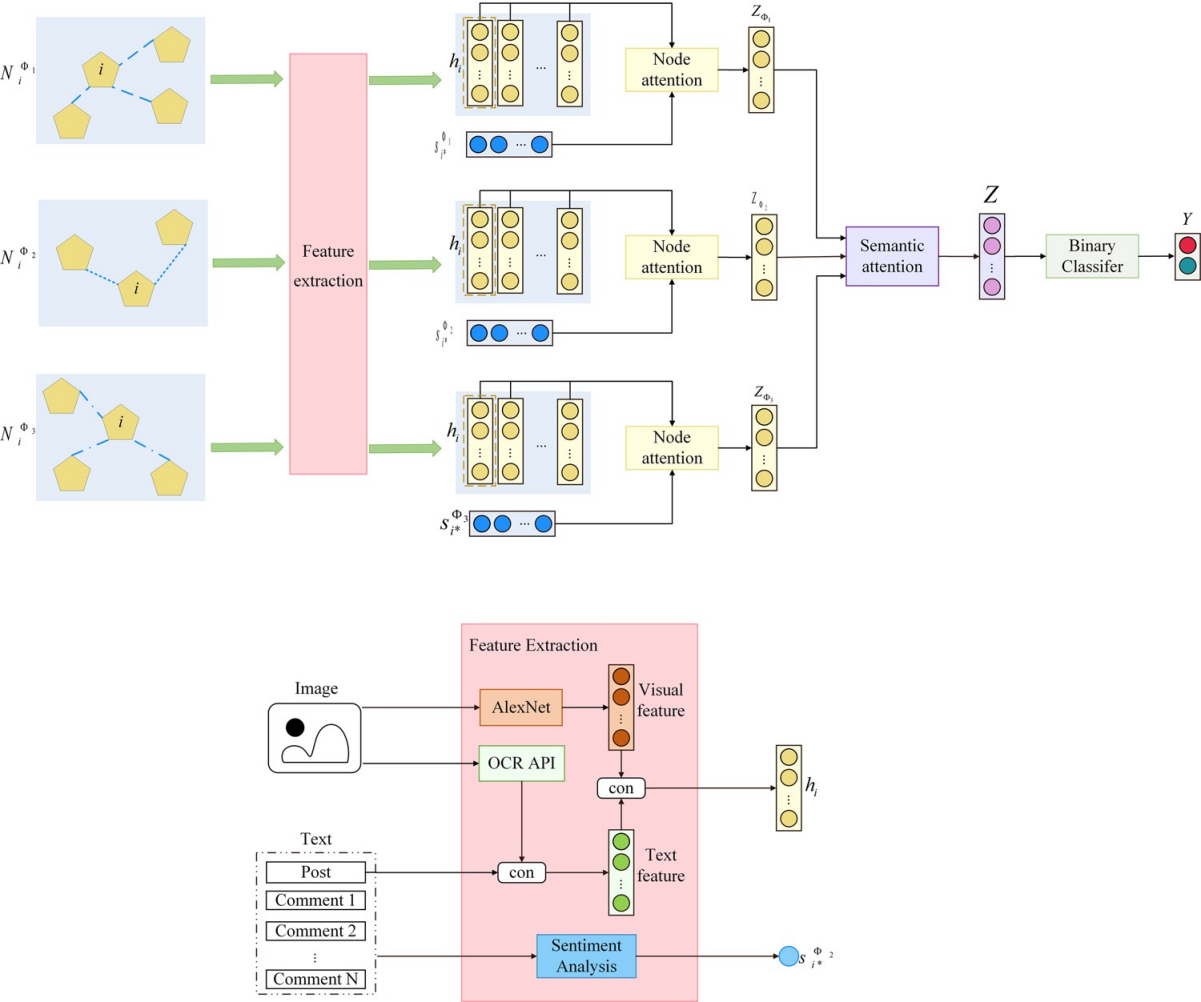
The overall framework of the MHAN. The upper part illustrates the overall structure of the MHAN. After feature extraction, the initial features of post nodes are processed by node-level attention, semantic-level attention, and classifier successively to acquire the final classification result Y. The procedure for feature extraction can be seen in the lower part. The initial features are extracted from posts’ appended visual and textual content.

In this study, an improved model for identifying microblog rumors named the Microblog-HAN (MHAN) is proposed. The overall structure of the proposed model is shown in Fig 2.

The appended pictures, text content, comment list, and neighbors of each post are fed to the MHAN input. The task of the first part of the proposed model is to extract the initial features of the post node and analyze the positions of comments. Since a post can include the content displayed in the form of images, graphical characters need to be recognized and attached to the end of the textual content, as these images containing rich text can supplement the post’s text. The text included in the images is extracted by a third-party optical character recognition API [29]. In addition to text identification, visual features of appended pictures are extracted by the AlexNet [30]. The initial feature *h*_*i*_ of post node *i* represents a concatenation of the text features acquired from the word2vec model and the image features calculated by the convolution layers. As for commenters’ standpoint, the SnowNLP is used for the text sentiment analysis. The weight between the post nodes obtained based on the meta-path PCUCP is set according to the average sentiment scores between the posts and comments.

As mentioned previously, the second part of the proposed model is an improved HAN that considers the meta-path-based weights. It aims at synthesizing the structure information of the heterogeneous microblog graph to generate the final embedding of post nodes. By analyzing the microblog posting historical data, it can be revealed that posts involving the same topic tend to have similar credibility, and meta-path PP can represent this relationship. From the perspective of users’ preferences, the behaviors of users follow relatively stable patterns. Namely, users who post microblog rumors generally have a notorious record of fabricating other rumors. In addition, regardless of whether the source channel is reliable or doubtful, news on a specific topic usually has a relatively steady audience. Although receivers’ ability to identify rumors can vary, they are more likely to take similar positions on the same type of posts. The above-mentioned two users’ behaviors can be represented by the meta-paths PUP and PCUCP.

In the proposed model, the meta-paths PUP, PCUCP, and PP in the heterogeneous microblog graph are selected to participate in model training, and denoted by Φ_1_, Φ_2_, and Φ_3_, respectively. After being processed by the node-level and semantic-level attention mechanisms, the initial features of post nodes are fully fused with the information of the meta-path-based neighbors and then input into the classifier for rumor detection.

#### 3.3.1. Node-level attention

In a heterogeneous graph, the meta-path-based neighbors of each node are of unequal importance. The node-level attention plays an essential role in learning the importance of every neighbor and aggregating important neighbors to form a new node embedding [8]. Suppose posts (i.e., nodes) *i* and *j* are linked by a meta-path Ф; thus, nodes *i* and *j* are neighbors based on the meta-path Ф. Assume the importance of node *j* to node *i* is denoted by *e*_*i*,*j*_^Φ^, which can be calculated by the self-attention mechanism. After the weighed softmax normalization of *e*_*i*,*j*_^Φ^, the attention weight coefficient *α*_*ij*_^*Φ*^ can be obtained by

$$e_{ij}^{\Phi }={\mathrm{att}}_{\mathrm{node}}(h_{i},h_{j};\Phi ),$$ (2)

$$\alpha_{ij}^{\Phi }=\mathrm{weighed}\;\mathrm{softmax}(e_{ij}^{\Phi })=\frac{\exp(\sigma (a_{\Phi }^{T}Wn[h_{i}\|h_{j}]))\times s_{ij}^{\Phi }}{\sum_{k\in N_{i}^{\Phi }}\exp(\sigma (a_{\Phi }^{T}Wn[h_{i}\|h_{k}]))\times s_{ik}^{\Phi }},$$ (3)

where att_node_ denotes the node-level attention deep neural network, whose parameters can be represented by *W*_*n*_ and *a*_Ф_; *a*_Ф_ denotes the node-level attention vector of a meta-path Ф, and it carries the specific semantic information on the meta-path Ф, which is shared among all nodes; $s_{ij}^{\Phi }$ is the weight obtained based on the meta-path Ф between the nodes *i* and *j*, guiding the computation of *α*_*ij*_^Φ^. More specifically, $s_{ij}^{\Phi_{1}}=s_{ij}^{\Phi_{3}}$, and $s_{ij}^{\Phi_{2}}$ is calculated by the average difference value of users’ stance on the posts *i* and *j*.

**Fig 3:**
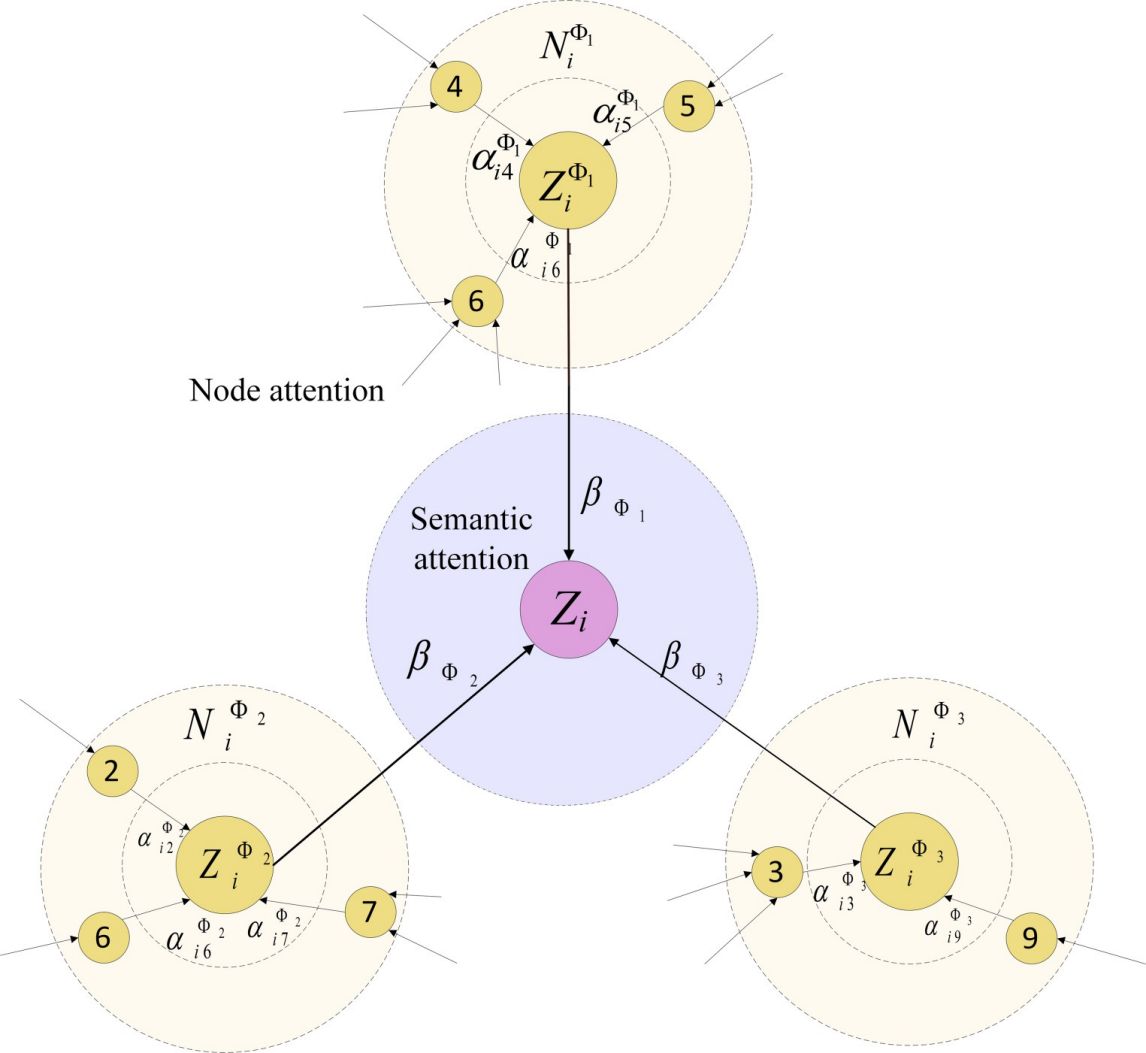
The hierarchical attention mechanism of the MHAN model. Nodes 4–6 are neighbors of node *i* based on meta-path Ф_1_; nodes 2, 6, and 7 are neighbors of node *i* based on meta-path Ф_2_; nodes 3 and 9 are neighbors of node *i* based on meta-path Ф_3_. After the aggregation on each meta-path, embeddings with different semantics are fused to obtain the final representation of node *i*, which is denoted as *Z*_*i*_.

The meta-path-based embedding of node *i* can be attained by aggregating the embedding of neighboring nodes with the corresponding weight coefficients. The aggregating process is depicted in Fig 3. To stabilize the learning process of self-attention, multi-head attention is included in the analysis. The node-level attention is performed *K* times independently, and the output features are concatenated to obtain the final meta-path-based node embedding of *I*, which is given by

$$z_{i}^{\Phi }=\overset{K}{\underset{K=1}{\|}}\sigma (\sum_{j\in N_{i}^{\Phi }}\alpha_{ij}^{\Phi }\cdot h_{j}).$$ (4)

#### 3.3.2. Semantic-level attention

Different meta-paths in a heterogeneous graph can imply different semantic information. The mission of semantic-level attention is to select the meaningful meta-paths and fuse semantics revealed by these meta-paths [8]. The node-level attention output includes three groups of node embeddings with specific semantics, which is denoted by $\left\{Z_{\Phi_{1}},Z_{\Phi_{2}},Z_{\Phi_{3}}\right\}$. However, one semantic-specific node embedding can reflect nodes only from one aspect. For instance, $Z_{\Phi_{1}}$ contains only the semantic information that the same user releases two posts, $Z_{\Phi_{2}}$ reflects that the posts are forwarded or commented by the same user, and $Z_{\Phi_{3}}$ indicates that the posts share a common topic. To aggregate these semantic-specific node embeddings into a more comprehensive one, semantic-level attention is used to learn the importance of each of the meta-paths $\left(w_{\Phi_{1}},w_{\Phi_{2}},w_{\Phi_{3}}\right)$. Similar to the node-level attention, the weight coefficient of each meta-path $\left(\beta_{\Phi_{1}},\beta_{\Phi_{2}},\beta_{\Phi_{3}}\right)$ represents the softmax normalization result of $\left(w_{\Phi_{1}},w_{\Phi_{2}},w_{\Phi_{3}}\right)$. The corresponding mathematical formulas in [8] are shown as follows:

$$(\beta_{\Phi_{1}},\beta_{\Phi_{2}},\beta_{\Phi_{3}})={\mathrm{att}}_{\mathrm{sem}}(Z_{\Phi_{1}},Z_{\Phi_{2}},Z_{\Phi_{3}}),$$ (5)

$$w_{\Phi_{i}}=\frac{1}{\left|V\right|}\sum_{i\in V}q^{T}\cdot \tanh(Ws\cdot z_{i}^{\Phi }+b),$$ (6)

$$\beta_{\Phi_{i}}=\frac{\exp(w_{\Phi_{i}})}{\sum_{j=1}^{3}\exp(w_{\Phi_{j}})},$$ (7)

where att_*sem*_ denotes the semantic-level attention deep neural network, and it can be formularized as Eqs (7) and (9); *W*_*s*_ is the weight matrix, *b* is the bias vector, and *V* is the set of all post nodes.

By averaging the nonlinear transformation of semantic-specific node embeddings and semantic-level attention vector *q*, the importance of meta-path Ф_*i*_, $w_{\Phi_{i}}$, is obtained.

With the learned weight coefficient $\left(\beta_{\Phi_{1}},\beta_{\Phi_{2}},\beta_{\Phi_{3}}\right)$, the semantic-specific node embeddings are fused as follows:

$$Z=\sum_{i=1}^{3}\beta_{\Phi_{i}}\cdot Z_{\Phi_{i}}.$$ (8)

As the final node embedding, *Z* aggregates the semantic information of meta-paths PUP, PCUCP, and PP, which are then input into the fully connected layer. The model is trained to minimize the cross-entropy loss function, which is given by

$$L=‐\sum_{i}\left[y_{i}\log(p_{i})+(1‐y_{i})\log(1‐p_{i})\right],$$ (9)

where *p*_*i*_ denotes the possibility of rumor class predicted by the model, and *y*_*i*_ denotes the ground truth where "1" represents a rumor, and "0" represents a non-rumor.

## 4. Experimental results

### 4.1 Datasets

The proposed MHAN was evaluated on two real Sina Weibo rumor datasets, Weibo2016 and Weibo2021. The Weibo2016 dataset was provided by Ma et al. [3] of Hong Kong Baptist University. It contained 4,664 labeled microblog posts. The rumors originated from the false information published by the Weibo Community Management Center before 2016. The Weibo2021 dataset consisted of 6,245 posts, and its rumor data were also acquired from the verified rumors published by the Weibo Community Management Center. The records of rumors and the corresponding comments and reposts from 2019 to 2021 were collected by the crawler. A similar number of non-rumor posts in the same period were also crawled to achieve a class balance. We made the Weibo2021 dataset publicly accessible. Please see *https*:*//github*.*com/lemon-coder/MicroblogHAN-dataset* for details. The statistics of the two datasets are given in Tables 1 and 2.

**Table 1:** Statistics of the Weibo2016 and Weibo2021 datasets.

Parameter	Weibo2016	Weibo2021
Posts	4,664	6,245
Non-rumors	2,351	3,162
Rumors	2,313	3,083
Users	2,746,818	253,691
Reposts and comments	3,805,656	315,218

**Table 2:** Statistics of the Rumors and Non-rumors in Weibo2016 and Weibo2021 datasets.

	Weibo2016		Weibo2021	
	Rumor	Non-rumor	Non-rumor	Non-rumor
Average reposts and comments	705.8	902.9	35.9	63.7
PUP	1,207	106,859	3,297	3,895
PCUCP	1,863,886	2,395,274	63,411	69,657
PP	5,87	1,333	1,811	25,192
Pictures	1,982	1,860	1,033	1,388

### 4.2 Experimental settings

All of the models were trained on an NVIDIA Tesla P100-16GB GPU. For the MHAN, the OCR API from the Baidu AI open platform was used, and the AlexNet with five groups of convolution operations for a single GPU was employed for subsequent experiments. The dimension of the initial feature vector of each post-node was 6,500. Eights heads were used for the node-level attention, and the dimension of the node embedding vector was 64. In the semantic-level attention, the number of the hidden units was set to 128, and the output embedding of each node was a 64-dimension vector. The trainable parameters were optimized by the Adam algorithm with an initial learning rate of 0.001. The maximum number of the training epochs was set to 600, but the training could end earlier if the validation loss was not lowered for the last 100 epochs. The overall data were split into the training, validation, and tests set according to the rate of 3:1:1. Please see S1 Appendix for the training process and computation complexity analysis of MHAN. Besides, the variation curve of the loss function and validation set accuracy were depicted in S1 and S2 Figs to illustrate the convergence process better. Four metrics were adopted to evaluate the performance of the models, namely, accuracy, precision, recall, and F1-score. The accuracy represents the recognition accuracy rate on all data; precision denotes the proportion of true positive samples among all positive samples predicted by the classifier; recall denotes the proportion of positive samples correctly predicted in the total positive samples; F1-score is the harmonic average of precision and recall.

First, the proposed MHAN model was compared with the other models on the Weibo2016 dataset. The experimental results are shown in Table 3; except for the MHAN model and its derivative models, the results of all models were provided by Ma et al. [3]. To validate the stability of MHAN, we also conducted the five-fold cross-validation analysis. The experimental results of the other four splits are shown in S1 Table.

**Table 3:** Experimental results on the Weibo2016 dataset.

Method	Class	Accuracy	Precision	Recall	F1-score
DTR	Rumor	0.732	0.738	0.715	0.726
	Non-rumor		0.726	0.749	0.737
DTC	Rumor	0.831	0.847	0.815	0.831
	Non-rumor		0.815	0.847	0.830
RFC	Rumor	0.849	0.786	0.959	0.864
	Non-rumor		0.947	0.739	0.830
SVM-RBF	Rumor	0.818	0.822	0.812	0.817
	Non-rumor		0.815	0.824	0.819
SVM-TS	Rumor	0.857	0.839	0.885	0.861
	Non-rumor		0.878	0.830	0.857
GRU	Rumor	0.910	0.876	0.956	0.914
	Non-rumor		0.952	0.864	0.906
PPC	Rumor	0.921	0.896	0.962	0.923
	Non-rumor		0.949	0.889	0.918
MHAN	Rumor	0.942	0.937	0.949	0.942
	Non-rumor		0.948	0.935	0.941
MHAN w/o PUP	Rumor	0.921	0.907	0.938	0.922
	Non-rumor		0.935	0.903	0.919
MHAN w/o PCUCP	Rumor	0.926	0.944	0.906	0.925
	Non-rumor		0.909	0.946	0.927
MHAN w/o PP	Rumor	0.932	0.930	0.936	0.933
	Non-rumor		0.935	0.929	0.932
MLP	Rumor	0.799	0.793	0.812	0.803
	Non-rumor		0.806	0.787	0.796

DTR [31]: a decision-tree-based model that ranks the clustered similar posts by their likelihood of being rumors.

DTC [10]: a decision-tree-based model that manually extracts features from the message, user, topic, and propagation.

RFC [1]: a random-forest-based model that uses a batch of handcrafted temporal features.

SVM-RBF [2]: an SVM-based model with the RBF kernel that uses Weibo-specific features.

SVM-TS [13]: an SVM-based model that uses the temporal characteristics of rumor features.

GRU [3]: a GRU-based model that learns continuous representations of microblog events.

PPC [17]: a model integrating the RNN and CNN.

MHAN w/o PUP: the MHAN model without the consideration of the meta-path PUP.

MHAN w/o PCUCP: the MHAN model without the consideration of the meta-path PCUCP.

MHAN w/o PP: the MHAN model without the consideration of the meta-path PP.

MLP: the MHAN model without the consideration of any meta-path, where the initial features of posts are directly input into the multilayer perception network.

Next, another experiment was conducted on the Weibo2021 dataset. As there was no available source code for the other models, only the MHAN and its derivative models were evaluated on this dataset, and their performances are given in Table 4. The five-fold cross-validation was also employed in the Weibo2021 dataset. The experimental results are shown in S2 Table.

**Table 4:** Experimental results on the Weibo2021 dataset.

Method	Class	Accuracy	Precision	Recall	F1-score
MHAN	Rumor	0.922	0.926	0.909	0.917
	Non-rumor		0.918	0.934	0.926
MHAN w/o PUP	Rumor	0.923	0.933	0.903	0.918
	Non-rumor		0.914	0.941	0.928
MHAN w/o PCUCP	Rumor	0.922	0.919	0.917	0.918
	Non-rumor		0.924	0.927	0.926
MHAN w/o PP	Rumor	0.905	0.896	0.906	0.901
	Non-rumor		0.913	0.905	0.909
MLP	Rumor	0.868	0.866	0.855	0.861
	Non-rumor		0.870	0.880	0.875

### 4.3 Result analysis

As shown in Table 3, the traditional machine learning models that rely on handcrafted features such as DTR, DTC, RFC, SVM-RDF, and SVM-TS, generally perform poorly on the Weibo2016 dataset, achieving an accuracy of less than 90% on the test set. This demonstrates that these models are not appropriate for rumor detection, as the handcraft features can reflect rumor characteristics only at a comparatively shallow level, lacking a higher-level representation.

The accuracy and F1-score of the GRU model on the test set are higher than those of the traditional machine learning classifiers. There are two main reasons for this phenomenon. First, as a neural network model, the GRU can automatically learn deep potential features, thus surpassing the traditional approaches. Second, the GRU can capture variations in contextual information of microblogs over time.

The PPC model outperforms the GRU model. This is because the PPC model combines variations from different levels after the propagation path is learned by the RNN and CNN, which is remarkably beneficial to rumor detection.

The best performance is achieved by the proposed MHAN model, whose accuracy on the test set reaches 94.2%. The higher recall on rumors suggests that MHAN is skilled in filtering rumors from mixed messages, whereas the greater precision on non-rumors imparts reliability to posts classified as true by MHAN. Besides, the highest F1-scores of MHAN on both rumors and non-rumors indicate its superior comprehensive classification capability. With the ideal interpretability, the MHAN uses the attention mechanism to fuse different semantics: "posted by the same user," "reposted by the same user," and "sharing the same topic." In this way, the structure information of the heterogeneous microblog graph is thoroughly mined, and users’ behavior patterns supplement the pure content of the post in need of identification. This is the main reason why MHAN has better performance than other existing methods.

The ablation studies further justify the above explanation. Compared with the MHAN model, the values of accuracy and F1-score of the MLP, MHAN w/o PUP, MHAN w/o PCUCP, and MHAN w/o PP are much lower. Thus, the semantics of all these three meta-paths are meaningful in the rumor recognition task, and the absence of any meta-path can adversely affect model performance.

The experimental results on the Weibo2021 dataset indicate that adding the meta-paths is beneficial to the rumor detection accuracy; namely, the performance of the MLP model is eclipsed by the other models. The results demonstrate that compared to the classification methods designed for isolated posts, the graph model performs better on social media tasks. The heterogeneous graph model with the node-level and semantic-level attention mechanisms could effectively learn the representations of posts using the structural information. Moreover, the effects of the three meta-paths on the rumor detection result differed. For instance, the MHAN w/o PP model performs the worst among all the models except for the MLP model in terms of nearly all metrics, so it could be concluded that the meta-path PP is the most significant effect among all meta-paths.

In contrast, the influences of the meta-paths PUP and PCUCP are relatively limited. As shown in Table 4, the accuracy of the MHAN model reaches 92.2%, which is comparable to that of the MHAN w/o PCUCP model but slightly lower than that of the MHAN w/o PUP model, whose accuracy is 92.3%. Moreover, the values of the F1-score of these models are close to each other, and they are all characterized by the lower recall of rumors and precision on non-rumors. These results demonstrate that the introduction of meta-path PUP or PCUCP slightly affects the detection result.

**Fig 4:**
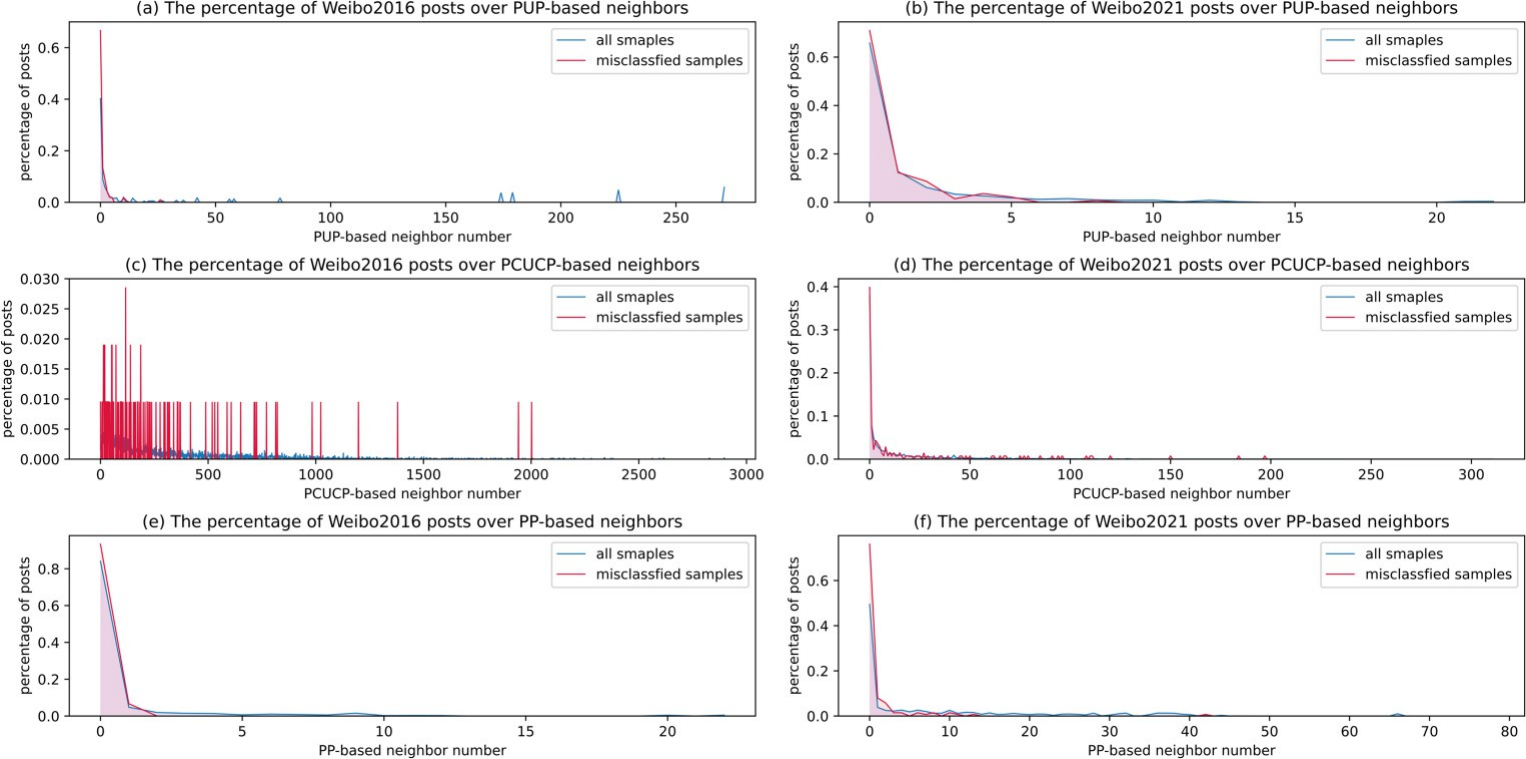
The percentage of posts over the number of 3 types of meta-path-based neighbors. The left column depicts how the proportion of posts changes in the Weibo2016 dataset, while the right column depicts that of the Weibo2021 dataset. The red line represents misclassified samples, and the blue line represents all samples.

To conduct error analysis, six line graphs were drawn in Fig 4 to delineate the percentage of incorrectly classified samples over the number of meta-path based neighbors, and also compare with the distribution of all samples. From Fig 4, we can conclude that whether in Weibo2016 or Weibo2021, compared with overall data distribution, the posts with fewer neighbors based on each meta path account for a higher proportion of the misclassified posts. The reason may lie in that the MHAN model is a graph-based model, calculating node embeddings through the features neighboring nodes. Suppose a post node connects to too few neighbors in the dataset. In that case, MHAN will fail to fully mine the corresponding user behaviors, and the effect of rumor classification will descend. In addition, some misclassified posts deeply rely on videos as the leading information carrier, which MHAN cannot process for now. Although some other posts only present the main information on pictures, these appended pictures are the primary information is purely visual rather than graphic characters, which requires even more advanced visual semantic analysis. The benefits and limitations of existing methods and MHAN are summarized in Table 5 for details.

**Table 5:** Summarisation of the benefits and limitations of existing methods and MHAN.

Parameter	Traditional machine learning	Deep learning	MHAN
Model	Random forest [1], SVM [2, 13], Decision tree [10, 11, 31], Kernel learning [12]	GRU [3], CNN [4], RNN [14–16], CNN-RNN [17], CNN-LSTM [18], GAN [19]	AlexNet+HAN
Accuracy	Relatively low	High	High
Microblog modeling	Sequence, Tree	Sequence,Tree	Graph
Feature extraction	Manual	Automatic	Automatic
Modality of extracted features	Single modal	Single modal, Multimodal	Multimodal
Requirement for training data	Easy to construct posts’ and users’ features	Large amount of training samples	Modeled as a highly connected graph
Convergence speed	Generally fast	Generally slow	Slow

### 4.4 Visualization of post nodes and attention coefficients

**Fig 5:**
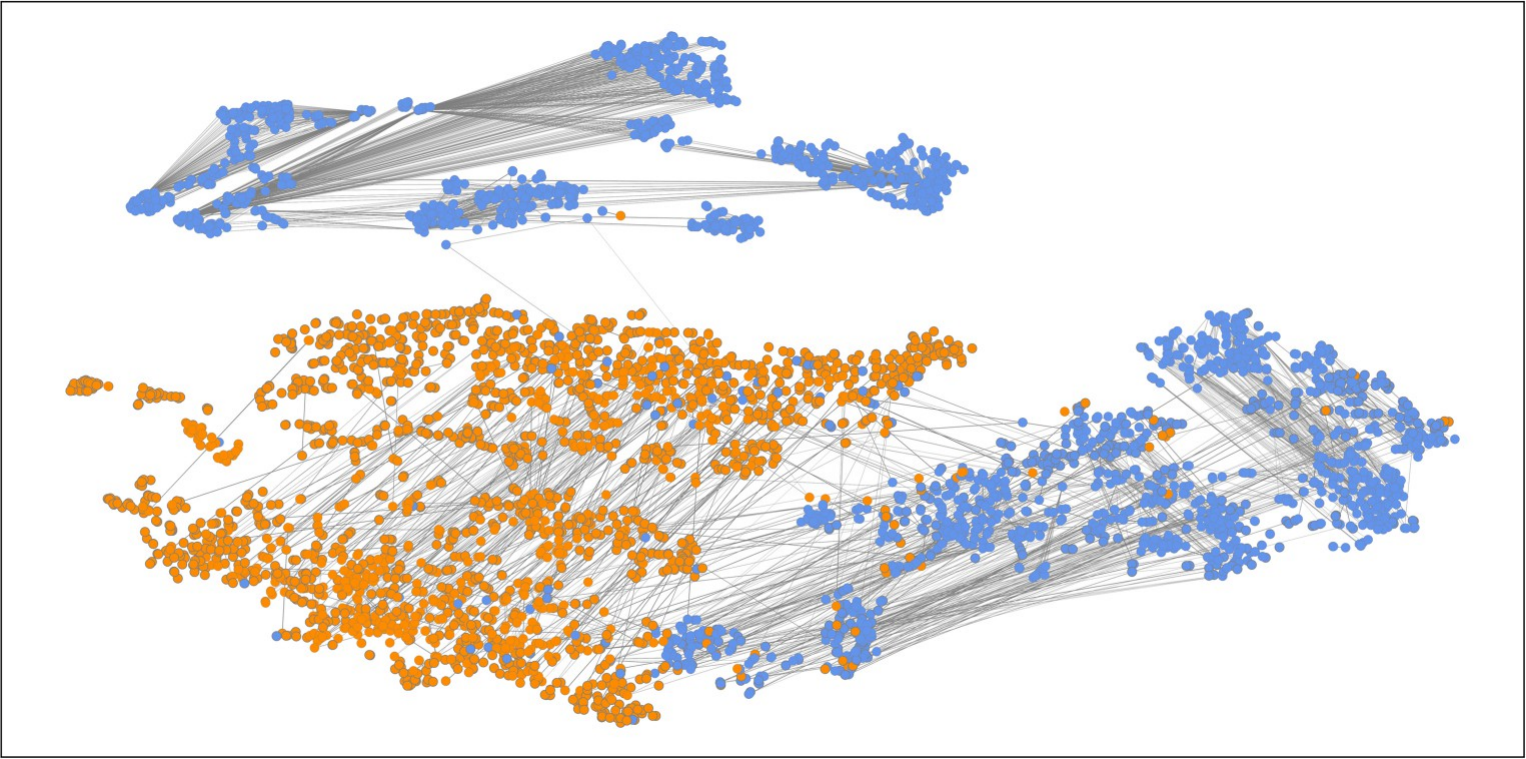
The visualization of the Weibo2016 posts and the weight coefficients of the meta-path PUP. The output node features obtained by the MHAN model’s attention layers are projected onto a two-dimensional plane, and the node color indicates the class of posts. The attention weight coefficients of the meta-path PUP between nodes are denoted by the edge thickness.

**Fig 6:**
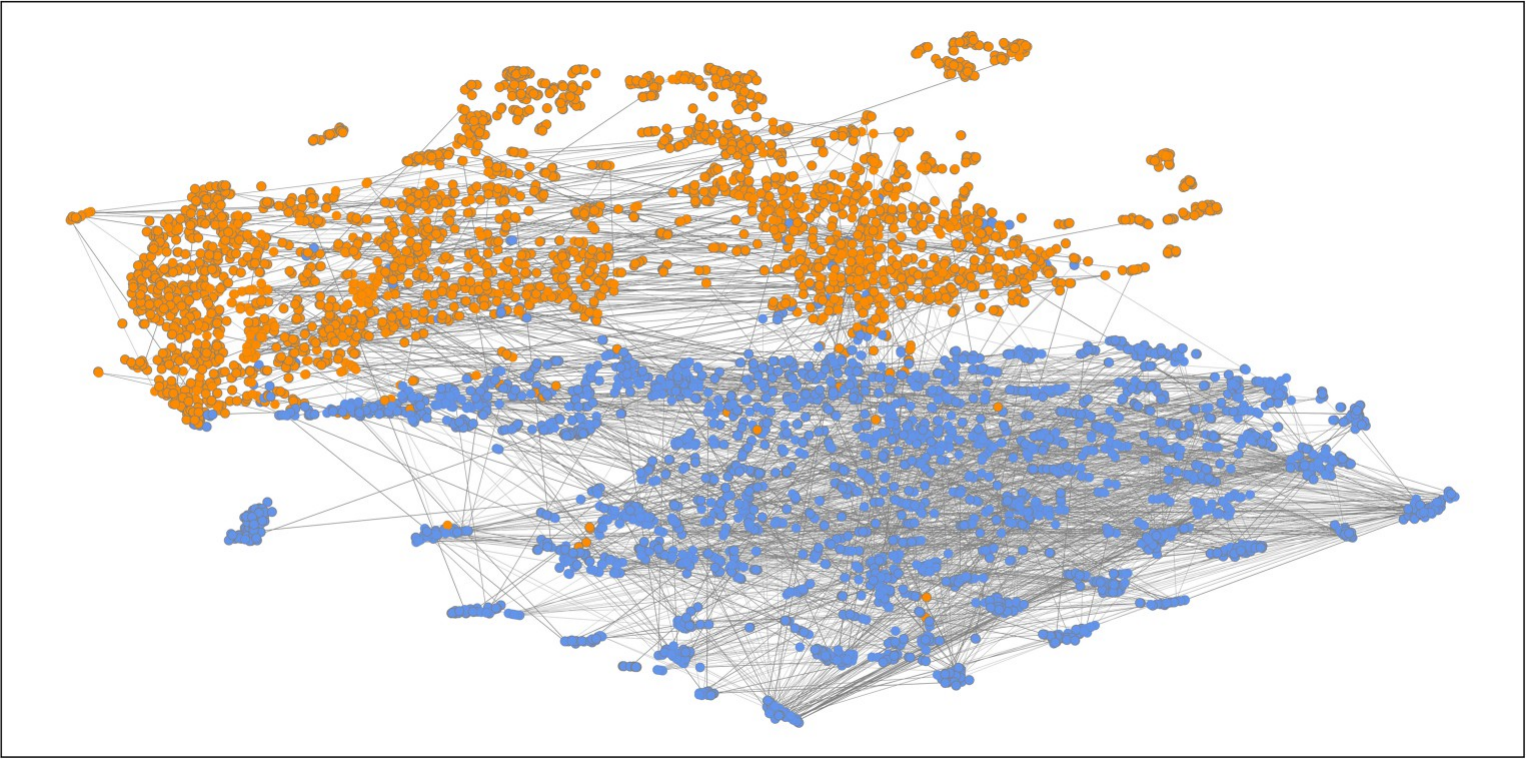
The visualization of the Weibo2021 posts and the weight coefficients of the meta-path PUP. The output node features obtained by the MHAN model’s attention layers are projected onto a two-dimensional plane, and the node color indicates the class of posts. The attention weight coefficients of the meta-path PUP between nodes are denoted by the edge thickness.

To test the classification results of the MHAN model on the Weibo2016 and Weibo2021 datasets further, the output feature representations computed by the MHAN model’s attention layers are mapped to a two-dimensional plane using the T-SNE algorithm. Specifically, Fig 5 (Fig 6) represents the visualization of the Weibo2016 (Weibo2021) posts and the weight coefficients of the meta-path PUP. Besides, we also draw the corresponding visualization of the Weibo2016 (Weibo2021) posts and the weight coefficients of the meta-paths PCUCP and PP in S3 Fig, respectively. The orange nodes represent microblog rumor posts, and the blue nodes denote posts with factual information.

In addition, the node-level attention weight coefficients of each meta-path are extracted and visualized in the form of node peripheries and edges. The thickness of the node periphery represents the importance of a microblog post to itself $\alpha_{ii}^{\Phi }$, while the edge thickness indicates the importance of two posts to each other. Because the calculation process of attention is asymmetric, that is, $\alpha_{ij}^{\Phi }\neq \alpha_{ji}^{\Phi }$, the correlation between two posts is measured by the mean of $\alpha_{ji}^{\Phi }$ and $\alpha_{ij}^{\Phi }$. To avoid the impact of excessive edges on the visualization result, the maximum degree of each node is limited to two.

As shown in Figs 5 and 6, and S3 Fig, the two types of nodes could be clearly distinguished by the proposed MHAN model, demonstrating its classification ability. From the perspective of connection, the edges between the same types of nodes are relatively dense, while the connections between different types of nodes are relatively sparse. In Fig 5 and S3(C) Fig, the attention coefficients of the meta-paths PUP and PP are visualized as edges between nodes. Based on the results presented in Fig 6 and S3(B) and S3(D) Fig, the weights of the meta-path PP are larger than those of the other meta-paths in the detecting rumor test on the Weibo2021 dataset. In S3(D) Fig, the post-nodes are the most scattered, but node clusters cover many short edges, which implies that the nodes sharing the same topic are more likely to be projected closer by the MHAN model.

## 5. Conclusion

In this study, an information dissemination network of a microblog is constructed as a heterogeneous graph, and a microblog rumor detection model named the MHAN is designed based on the AlexNet and HAN. Unlike the existing rumor detection models, in the proposed model, textual features of posts and visual features of appended pictures are used for rumor recognition. In addition, the proposed MHAN model uses a hierarchical attention mechanism to recognize different aggregate semantics revealed by the information diffusion network instead of identifying a single post. The experimental results on two rumor datasets show that the proposed MHAN can outperform the other models in the rumor classification task.

In future work, the information on videos and users could be combined to extract a more comprehensive initial representation of a post. In addition, the instance detection method could be used to obtain more precise information on the comments’ intentions. Finally, further investigation on the selection of meta-paths beneficial to rumor detection could be conducted.

## Supporting information

S1 Appendix The training process and computation complexity analysis of MHAN. (PDF)

S1 Fig The variation curve of accuracy and loss function of MHAN during the training process on the Weibo2016 dataset. (TIF)

S2 Fig The variation curve of accuracy and loss function of MHAN during the training process on the Weibo2021 dataset. (TIF)

S3 Fig The visualization of the posts and the weight coefficients of the meta-path PCUCP and PP in the Weibo2016 and Weibo2021 datasets. The output node features obtained by the MHAN model’s attention layers are projected onto a two-dimensional plane; the node color indicates the class of posts. The attention weight coefficients of the meta-paths between nodes are denoted by the edge thickness.
(TIF)

S1 Table Cross-validation experimental results on the Weibo2016 dataset. (PDF)

S2 Table Cross-validation experimental results on the Weibo2021 dataset. (PDF)

S3 Table The experimental results on Weibo2022 datasets to testify the universality of the MHAN model. (PDF)

S1 File (ZIP)
