# Supplementary material for: Microblog-HAN: A micro-blog rumor detection model based on heterogeneous graph attention network
Source: PLoS One. 2022 Apr 12;17(4):e0266598. doi: 10.1371/journal.pone.0266598 (PMC9004763; doi:10.1371/journal.pone.0266598)
Supplement: S1 Appendix — (PDF) [file pone.0266598.s001.pdf]

S1 Appendix

MHAN model is composed of AlexNet and HAN. The main body of HAN is hierarchical attention, node-level, and semantic-level attention. Denote n as the number of posts, m as the number of meta-paths, d as the dimension of the initial embedding of a post node. The time complexity of node-level attention can be represented as $O(mn^2d)$, and the complexity of semantic-level attention is $O(mnd)$. In terms of AlexNet, the time complexity of each layer can be denoted by $O(C_{in}C_{out}F^2K^2)$, where C_{in} is the number of the input channel, C_{out} is the output channel, F is the size of input feature maps, and K is the size of kernels. The total time complexity of ALexNet is the sum of that of each layer.

Like most neural networks, the massive time expense on the MHAN training process can never be overlooked. The variation curve of the loss function and validation set accuracy are depicted in S1 FigA1 and S2 FigA2 to illustrate the convergence process better. It is noticeable that MHAN converges rapidly in the first 50 epochs.
