# Supplementary figures and images for: Microblog-HAN: A micro-blog rumor detection model based on heterogeneous graph attention network
Source: PLoS One. 2022 Apr 12;17(4):e0266598. doi: 10.1371/journal.pone.0266598 (PMC9004763; doi:10.1371/journal.pone.0266598)

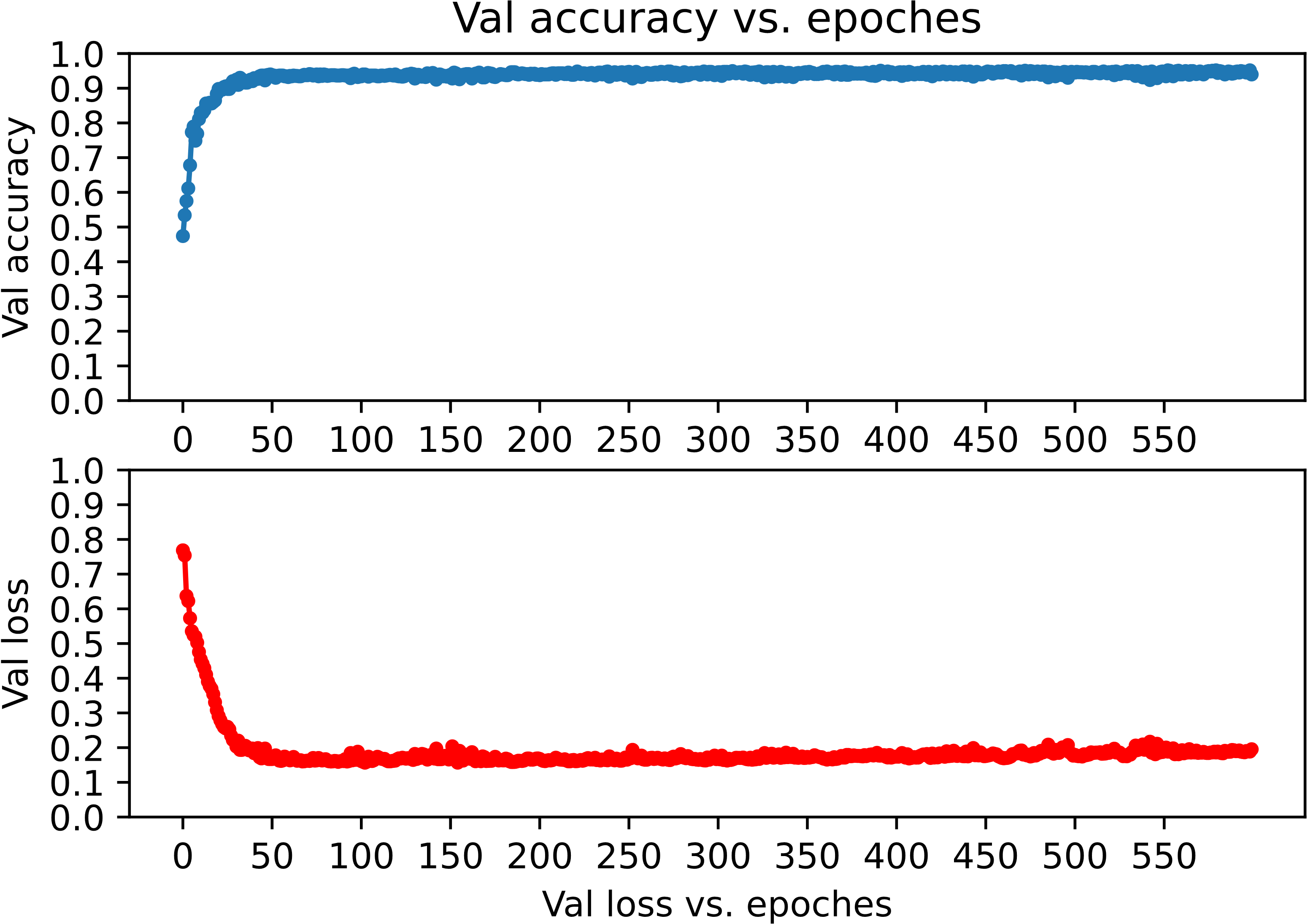

Supplement: S1 Fig — (TIF) [file pone.0266598.s002.tif]

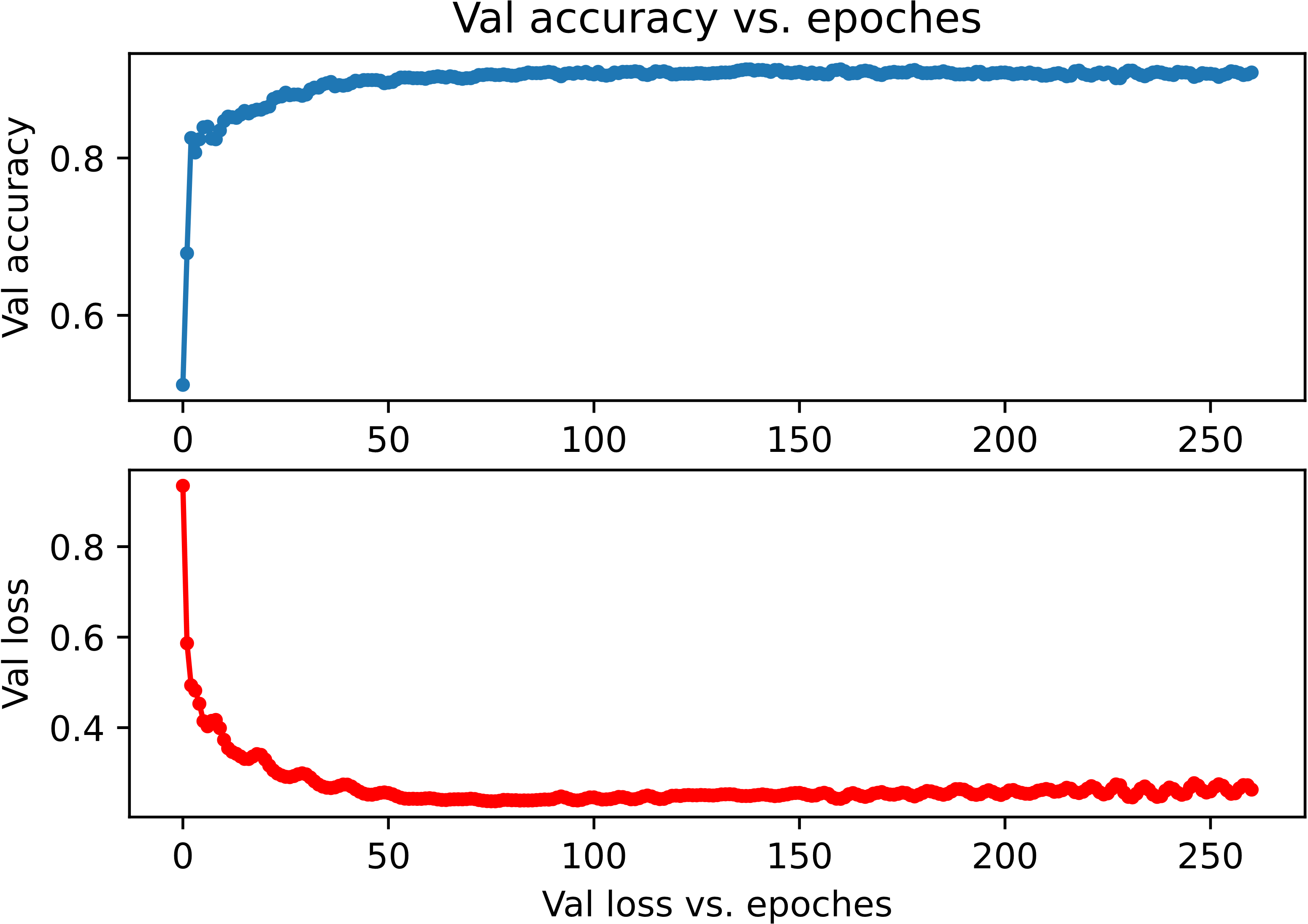

Supplement: S2 Fig — (TIF) [file pone.0266598.s003.tif]

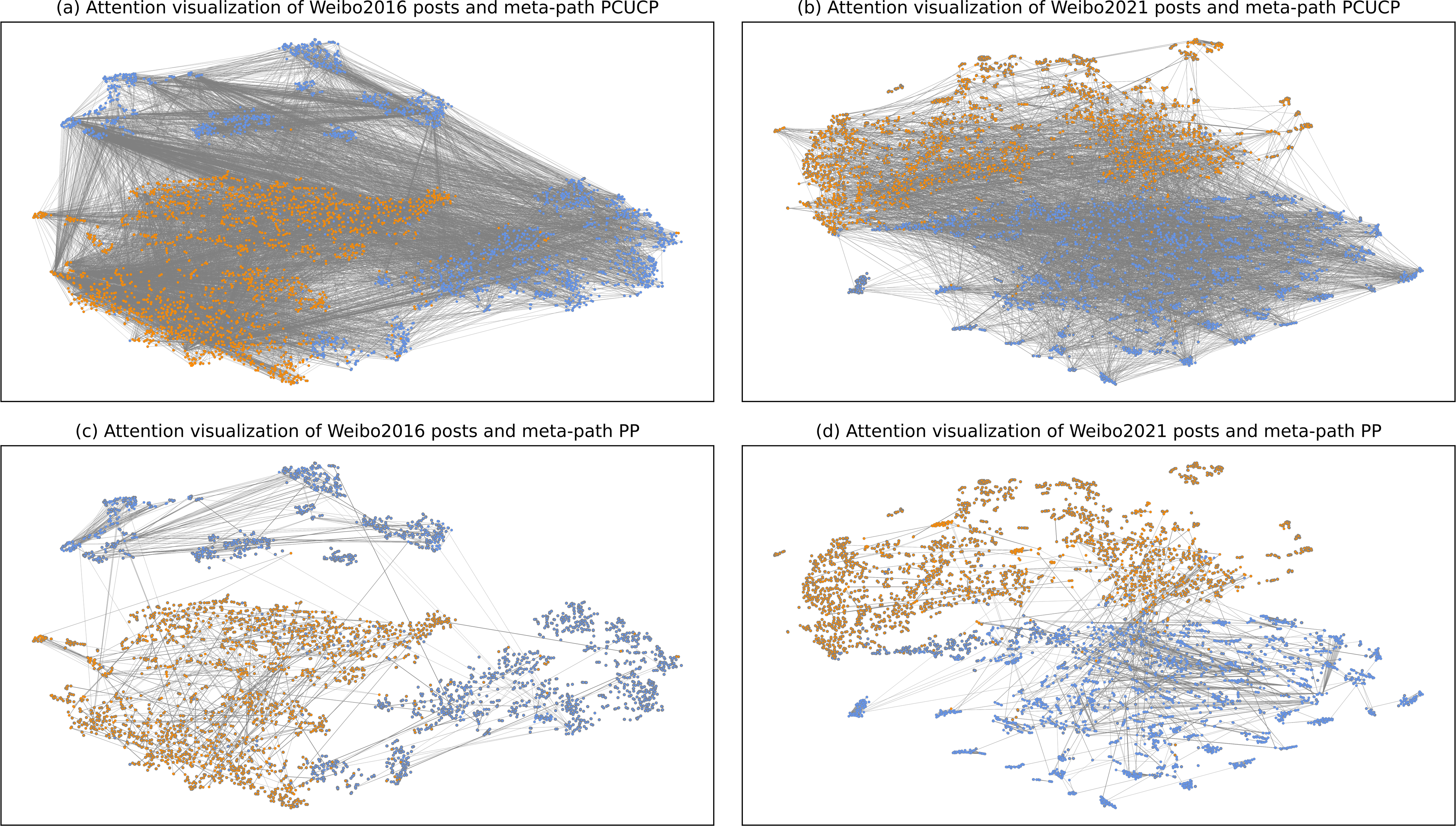

Supplement: S3 Fig — The output node features obtained by the MHAN model’s attention layers are projected onto a two-dimensional plane; the node color indicates the class of posts. The attention weight coefficients of the meta-paths between nodes are denoted by the edge thickness. (TIF) [file pone.0266598.s004.tif]

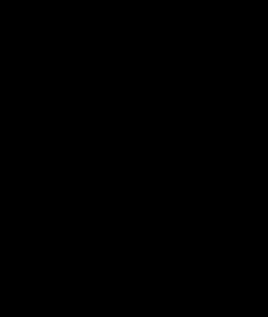

Supplement: S1 File — (ZIP) [file pone.0266598.s008.zip › code/data_preprocess/black.jpg]
