# Supplementary material for: Microblog-HAN: A micro-blog rumor detection model based on heterogeneous graph attention network
Source: PLoS One. 2022 Apr 12;17(4):e0266598. doi: 10.1371/journal.pone.0266598 (PMC9004763; doi:10.1371/journal.pone.0266598)
Supplement: S1 Table — (PDF) [file pone.0266598.s005.pdf]

**S1 Table**

Table A1 Cross-validation experimental results on the Weibo2016 dataset

| Method | Class | Accuracy | Precision | Recall | F1-score |
|---|---|---|---|---|---|
| Panel A Split1 | | | | | |
| MHAN | Rumor | 0.945 | 0.937 | 0.948 | 0.943 |
| | Non-rumor | | 0.953 | 0.943 | 0.948 |
| MHAN w/o PUP | Rumor | 0.936 | 0.947 | 0.916 | 0.931 |
| | Non-rumor | | 0.927 | 0.953 | 0.940 |
| MHAN w/o PCUCP | Rumor | 0.931 | 0.938 | 0.916 | 0.927 |
| | Non-rumor | | 0.926 | 0.945 | 0.935 |
| MHAN w/o PP | Rumor | 0.934 | 0.944 | 0.916 | 0.930 |
| | Non-rumor | | 0.926 | 0.951 | 0.939 |
| MLP | Rumor | 0.799 | 0.787 | 0.792 | 0.789 |
| | Non-rumor | | 0.811 | 0.806 | 0.809 |
| Panel B Split2 | | | | | |
| Method | Class | Accuracy | Precision | Recall | F1-score |
| MHAN | Rumor | 0.933 | 0.958 | 0.909 | 0.933 |
| | Non-rumor | | 0.911 | 0.958 | 0.934 |
| MHAN w/o PUP | Rumor | 0.911 | 0.905 | 0.922 | 0.913 |
| | Non-rumor | | 0.917 | 0.899 | 0.908 |
| MHAN w/o PCUCP | Rumor | 0.914 | 0.948 | 0.880 | 0.913 |
| | Non-rumor | | 0.884 | 0.950 | 0.916 |
| MHAN w/o PP | Rumor | 0.931 | 0.950 | 0.914 | 0.931 |
| | Non-rumor | | 0.914 | 0.950 | 0.931 |
| MLP | Rumor | 0.778 | 0.812 | 0.735 | 0.771 |
| | Non-rumor | | 0.749 | 0.823 | 0.784 |
| Panel C Split3 | | | | | |
| Method | Class | Accuracy | Precision | Recall | F1-score |
| MHAN | Rumor | 0.946 | 0.938 | 0.954 | 0.946 |
| | Non-rumor | | 0.955 | 0.939 | 0.947 |
| MHAN w/o PUP | Rumor | 0.924 | 0.904 | 0.945 | 0.924 |
| | Non-rumor | | 0.945 | 0.903 | 0.923 |
| MHAN w/o PCUCP | Rumor | 0.938 | 0.948 | 0.923 | 0.936 |
| | Non-rumor | | 0.928 | 0.951 | 0.940 |
| MHAN w/o PP | Rumor | 0.943 | 0.934 | 0.952 | 0.943 |
| | Non-rumor | | 0.953 | 0.935 | 0.944 |
| MLP | Rumor | 0.804 | 0.792 | 0.814 | 0.803 |
| | Non-rumor | | 0.816 | 0.793 | 0.804 |
| Panel D Split4 | | | | | |

| Method | Class | Accuracy | Precision | Recall | F1-score |
| --- | --- | --- | --- | --- | --- |
| MHAN | Rumor | 0.941 | 0.938 | 0.944 | 0.941 |
| | Non-rumor | | 0.944 | 0.938 | 0.941 |
| MHAN w/o PUP | Rumor | 0.939 | 0.924 | 0.957 | 0.940 |
| | Non-rumor | | 0.955 | 0.920 | 0.937 |
| MHAN w/o PCUCP | Rumor | 0.916 | 0.931 | 0.900 | 0.915 |
| | Non-rumor | | 0.902 | 0.933 | 0.917 |
| MHAN w/o PP | Rumor | 0.941 | 0.930 | 0.956 | 0.942 |
| | Non-rumor | | 0.953 | 0.927 | 0.940 |
| MLP | Rumor | 0.842 | 0.876 | 0.799 | 0.836 |
| | Non-rumor | | 0.814 | 0.886 | 0.848 |
