# Supplementary material for: Microblog-HAN: A micro-blog rumor detection model based on heterogeneous graph attention network
Source: PLoS One. 2022 Apr 12;17(4):e0266598. doi: 10.1371/journal.pone.0266598 (PMC9004763; doi:10.1371/journal.pone.0266598)
Supplement: S2 Table — (PDF) [file pone.0266598.s006.pdf]

S2 Table

Table A2 Cross-validation experimental results on the Weibo2021 dataset

Method	Class	Accuracy	Precision	Recall	F1-score
Panel A Split1					
MHAN	Rumor	0.920	0.949	0.888	0.917
	Non-rumor		0.894	0.952	0.922
MHAN w/o PUP	Rumor	0.929	0.951	0.904	0.927
	Non-rumor		0.901	0.953	0.930
MHAN w/o PCUCP	Rumor	0.922	0.947	0.893	0.919
	Non-rumor		0.898	0.950	0.924
MHAN w/o PP	Rumor	0.902	0.899	0.907	0.903
	Non-rumor		0.906	0.897	0.902
MLP	Rumor	0.861	0.877	0.842	0.859
	Non-rumor		0.847	0.881	0.864
Panel B Split2					
Method	Class	Accuracy	Precision	Recall	F1-score
MHAN	Rumor	0.913	0.920	0.900	0.910
	Non-rumor		0.906	0.925	0.916
MHAN w/o PUP	Rumor	0.916	0.929	0.897	0.912
	Non-rumor		0.905	0.934	0.919
MHAN w/o PCUCP	Rumor	0.905	0.914	0.889	0.901
	Non-rumor		0.896	0.920	0.908
MHAN w/o PP	Rumor	0.877	0.896	0.846	0.870
	Non-rumor		0.860	0.906	0.883
MLP	Rumor	0.853	0.859	0.836	0.847
	Non-rumor		0.847	0.869	0.859
Panel C Split3					
Method	Class	Accuracy	Precision	Recall	F1-score
MHAN	Rumor	0.926	0.951	0.894	0.922
	Non-rumor		0.903	0.956	0.929
MHAN w/o PUP	Rumor	0.917	0.947	0.881	0.913

	Non-rumor		0.892	0.953	0.922
MHAN w/o PCUCP	Rumor	0.924	0.950	0.893	0.920
	Non-rumor		0.902	0.954	0.927
MHAN w/o PP	Rumor	0.904	0.916	0.886	0.901
	Non-rumor		0.893	0.921	0.907
MLP	Rumor	0.884	0.909	0.849	0.878
	Non-rumor		0.862	0.918	0.889

Panel D　Split4

Method	Class	Accuracy	Precision	Recall	F1-score
MHAN	Rumor	0.927	0.953	0.901	0.926
	Non-rumor		0.903	0.954	0.928
MHAN w/o PUP	Rumor	0.925	0.952	0.898	0.924
	Non-rumor		0.900	0.953	0.926
MHAN w/o PCUCP	Rumor	0.926	0.950	0.901	0.925
	Non-rumor		0.902	0.951	0.926
MHAN w/o PP	Rumor	0.921	0.944	0.898	0.920
	Non-rumor		0.899	0.944	0.921
MLP	Rumor	0.886	0.910	0.860	0.884
	Non-rumor		0.863	0.912	0.887
