# Supplementary material for: Microblog-HAN: A micro-blog rumor detection model based on heterogeneous graph attention network
Source: PLoS One. 2022 Apr 12;17(4):e0266598. doi: 10.1371/journal.pone.0266598 (PMC9004763; doi:10.1371/journal.pone.0266598)
Supplement: S3 Table — (PDF) [file pone.0266598.s007.pdf]

S3 Table

This study mainly discussed experimental results on two datasets. For the evaluation of results, more recent data were crawled to testify the universality of the MHAN model. A total of 249 posts certified as rumors since January 1st, 2022, were collected and mixed with the Weibo2021 dataset to form the Weibo2022 dataset. The new dataset consists of 33328 comments and reposts. Following the previous experimental settings, the MHAN model and the derivative models were evaluated on Weibo2022, and the experimental results can be seen in Table A3.

Table A3 Experimental results on Weibo2022 datasets

Method	Class	Accuracy	Precision	Recall	F1-score
MHAN	Rumor	0.930	0.948	0.916	0.932
	Non-rumor		0.911	0.945	0.928
MHAN w/o PUP	Rumor	0.922	0.949	0.900	0.924
	Non-rumor		0.896	0.947	0.921
MHAN w/o PCUCP	Rumor	0.928	0.943	0.918	0.930
	Non-rumor		0.912	0.939	0.925
MHAN w/o PP	Rumor	0.899	0.917	0.888	0.902
	Non-rumor		0.881	0.911	0.896
MLP	Rumor	0.865	0.872	0.869	0.871
	Non-rumor		0.857	0.861	0.859
